# Supplementary figures and images for: Prolonged B-Lymphocyte-Mediated Immune and Inflammatory Responses to Tuberculosis Infection in the Lungs of TB-Resistant Mice
Source: Int J Mol Sci. 2023 Jan 6;24(2):1140. doi: 10.3390/ijms24021140 (PMC9861759; doi:10.3390/ijms24021140)

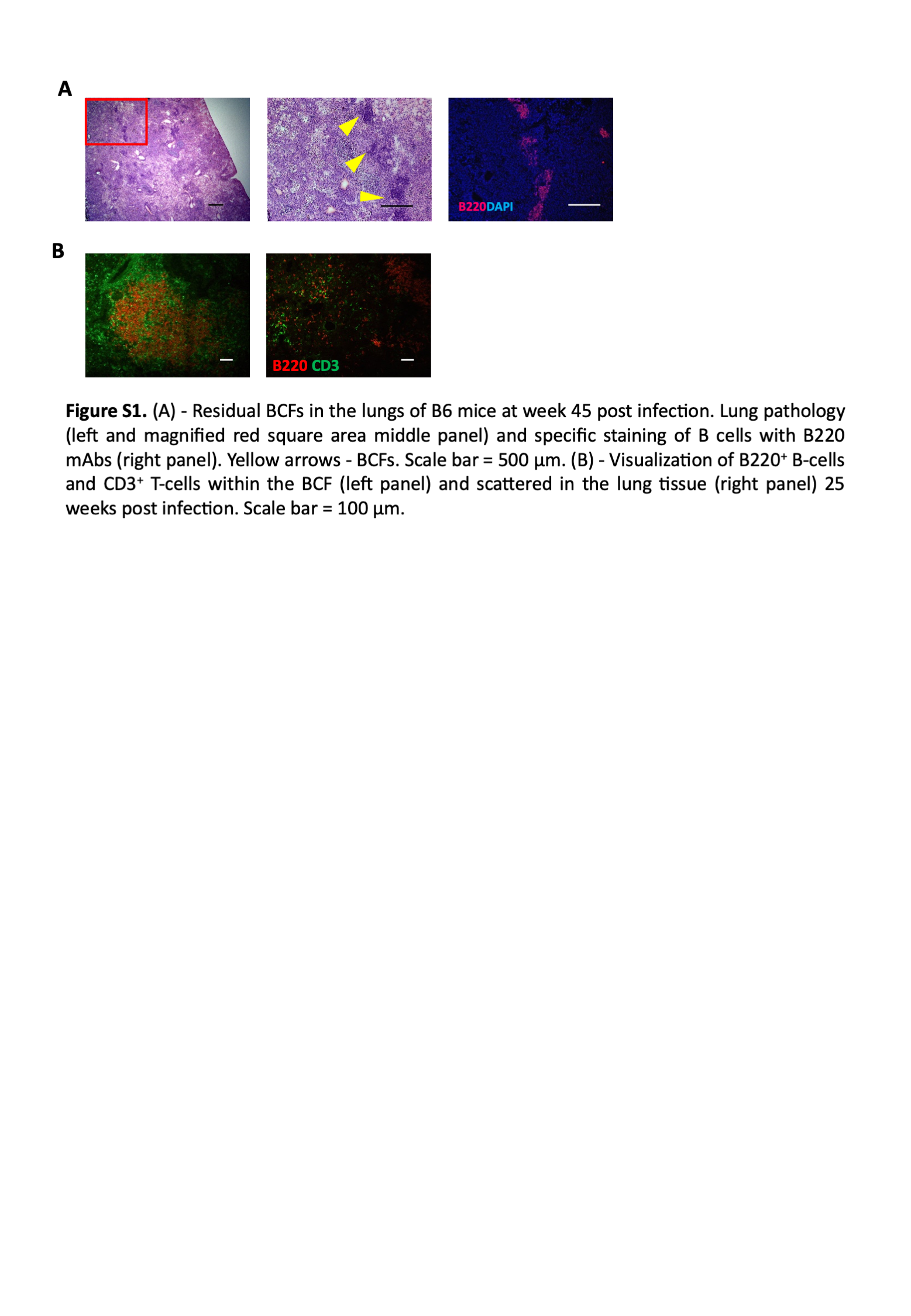

Supplement: Supplementary file 1 [file ijms-24-01140-s001.zip › Figure S1 revised.tiff]

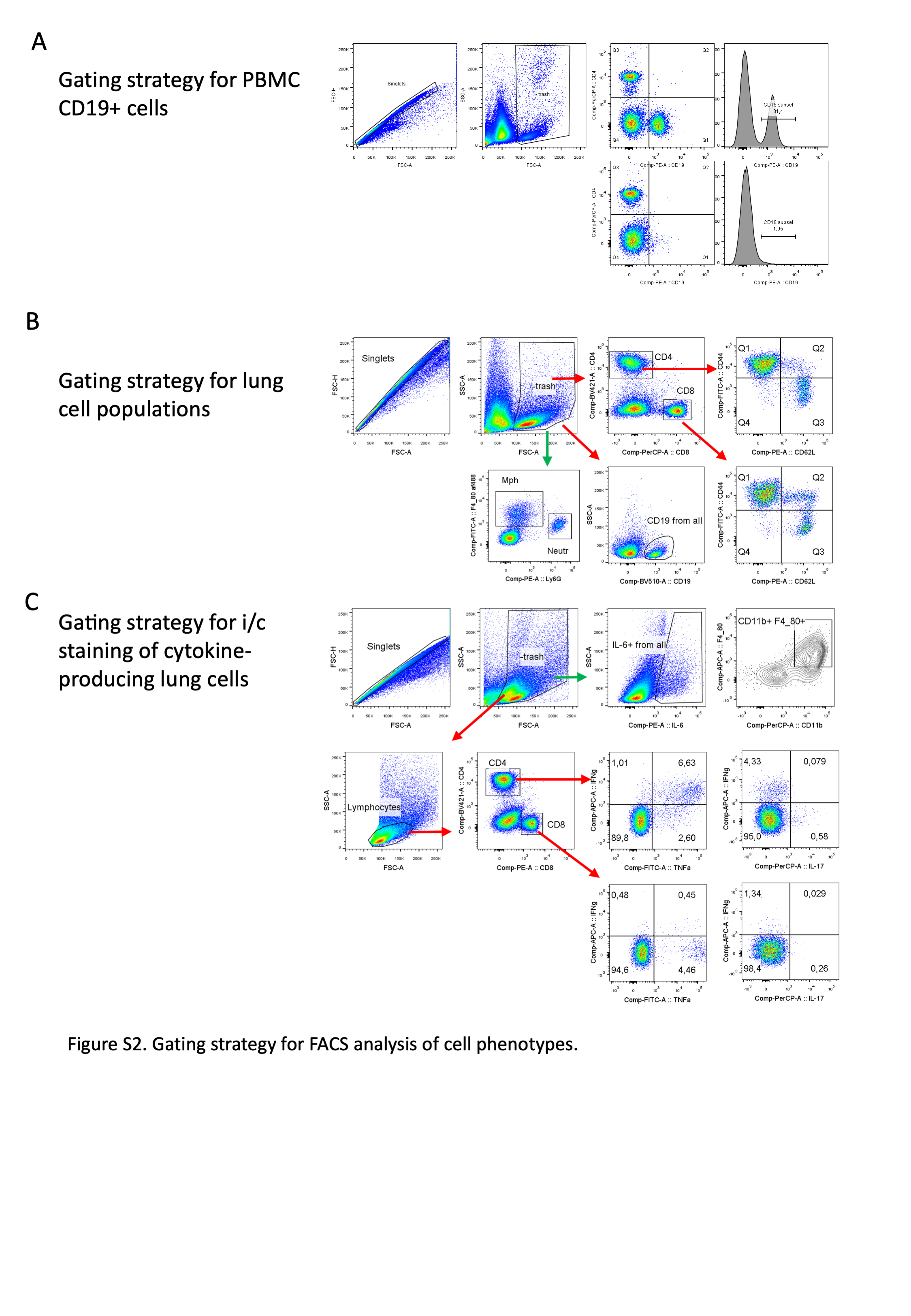

Supplement: Supplementary file 1 [file ijms-24-01140-s001.zip › Figure S2.tiff]

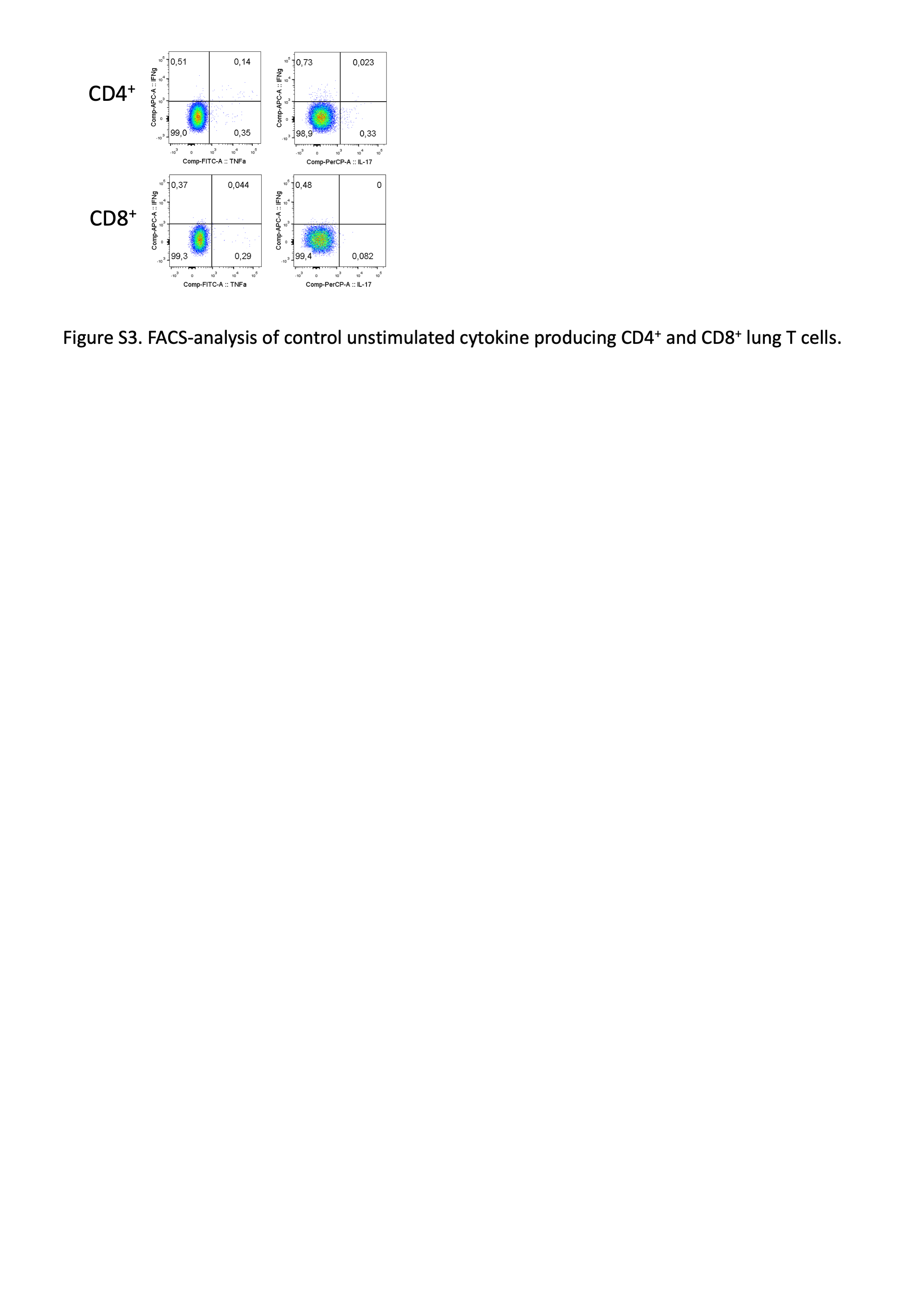

Supplement: Supplementary file 1 [file ijms-24-01140-s001.zip › Figure S3.tiff]
